# Activities of human amniotic membrane extracellular vesicles with a focus on ophthalmology

**DOI:** 10.1007/s11033-026-12756-0

**Published:** 2026-09-18

**Authors:** Matteo Lulli, Sara Calcagno, Silvia Peppicelli, Giulia Isoldi, Indu-Pal Kaur, Laura Papucci, Lucia Magnelli, Antonella Mannini, Nicola Schiavone

**Affiliations:** 1 Department of Experimental and Clinical Biomedical Sciences ‘Mario Serio’, University of Florence, Florence, Italy; 2 UGC-Centre of Advanced Study, University Institute of Pharmaceutical Sciences, Panjab University, Chandigarh, India; 3 Department of Experimental and Clinical Medicine, University of Florence. Largo Brambilla, 3 - 50134 Firenze, Italy

**Keywords:** Human Amniotic Membrane, hAM, Extracellular Vesicles, EV, Ophthalmology

## Abstract

For more than a century, the human amniotic membrane (hAM) has been recognized as an optimal tool for skin and corneal wound healing. It has attracted considerable attention from researchers in regenerative medicine due to its structural robustness as a scaffold and its content of epithelial and mesenchymal stromal cells, which exhibit low immunogenicity and immunotoxicity while retaining stem cell potential. Moreover, hAM possesses a range of therapeutic properties relevant to the treatment of various diseased tissues. These effects are mediated by extracellular components, including secreted factors, matrix elements, and extracellular vesicles (EVs). Despite this, a detailed characterization of the extracellular vesicles fraction of hAM, particularly regarding its molecular content and functional properties, remains limited. In this minireview, we summarize the available literature with particular attention to ophthalmology. Recent studies suggest that hAM-derived EVs may provide neuroprotective, anti-inflammatory, and pro-regenerative effects, supporting tissue repair and homeostasis in ocular pathologies. Understanding the molecular cargo of these vesicles, including proteins, miRNAs, and signaling molecules, could help unlock their therapeutic potential and guide the development of standardized cell-free treatments. Taken together, hAM-derived EVs represent a compelling cell-free therapeutic platform for ophthalmology, recapitulating many of the beneficial actions of the native tissue. However, clinical translation requires overcoming critical hurdles. We highlight these unresolved challenges, including manufacturing standardization, potency assessment, and reproducibility, while outlining the key safety and regulatory considerations essential for future ocular therapies.

## Introduction

The human amniotic membrane (hAM) is a specialized tissue involved in embryonic protection, isolation, and regulation. Structurally, it is relatively simple, consisting of a leaflet with a thickness ranging from 20 to 500 μm, composed of multiple stromal layers containing mesenchymal cells and internally limited by a single-cell epithelial layer [1]. hAM was first used in the first half of the last century to repair skin and corneal wounds [2, 3]. The interest in hAM resurged toward the end of the century, driven by advances in regenerative medicine, as it offers an optimal scaffold for synthetic tissue engineering and serves as a rich source of epithelial and mesenchymal stromal cells with immunomodulatory and regenerative properties that hold promise for therapeutic applications [1]. The hAM exhibits a broad spectrum of biological properties, including anti-fibrotic, anti-angiogenic, anti-apoptotic, anti-inflammatory, pro-regenerative, and pro-apoptotic effects, as well as low immunogenicity and immunotoxicity [4]. Different cells and extracellular components play distinct roles in these properties, depending on the physiological and/or experimental context [5].

It is known that the hAM secretome is characterized by an extracellular vesicle (EV) fraction and an EV-free fraction, each with distinct properties. Until now, the secretome has been studied mainly with respect to its functional effects and, predominantly, as a whole [6, 7]. Moreover, studies specifically addressing the composition of the hAM EV fraction remain limited, particularly considering that its content changes under different microenvironmental conditions, including those characterizing different physiological states of pregnancy [8].

In ophthalmology, beyond its established application in corneal wound repair, hAM has more recently been employed in the treatment of macular holes, retinal detachment, and other retinal pathologies [9, 10]. In this review, we present an overview of the current knowledge on the EV fraction of hAM, with a particular focus on its emerging applications in ophthalmology, contextualized within the broader properties and functions of the amniotic membrane.

This article is a narrative mini-review aimed at critically summarizing the current knowledge regarding EVs derived from hAM, with particular emphasis on their potential applications in ophthalmology. The literature discussed was identified through targeted searches of PubMed and other relevant databases using combinations of the terms “human amniotic membrane”, “extracellular vesicles”, “exosomes”, “microvesicles”, and “ophthalmology”. Given the limited number and heterogeneity of available studies, a formal systematic review approach was not applied; instead, emphasis was placed on identifying key findings, methodological limitations, and unresolved questions relevant to clinical translation.

## EVs: advantages over entire-cell/tissue treatment

EVs are a heterogeneous group of membrane-delimited structures secreted by virtually all cell types, playing essential roles in intercellular communication. Due to their characteristics and methodological limits, standardization in their isolation, handling, and nomenclature is mandatory. The “Minimal Information for Studies of Extracellular Vesicles” (MISEV 2023) guidelines, issued by the International Society for Extracellular Vesicles (ISEV), provide an ever-updated framework for researchers to improve the robustness of their data [11]. Historically, EV populations have been classified according to size, molecular characteristics, and proposed biogenesis pathways, leading to terms such as “exosomes”, “microvesicles” or “ectosomes”, and “apoptotic bodies” [12]. However, as emphasized by the MISEV 2023 guidelines, these terms should be applied cautiously because biogenesis cannot generally be inferred solely from EV size or commonly used molecular markers. Therefore, throughout this review, we preferentially use the generic term “EVs”, unless specific terminology is required when discussing previous studies. Different EV populations may display molecular signatures associated with their cellular origin and biogenesis pathways; however, such markers should not be considered definitive evidence of EV subtype identity. The MISEV 2023 guidelines suggest caution when using some operational size-related terms, the assessment of which can be method-dependent, or biogenesis-related terms, unless the EV origin can be securely assessed. Some terms are even discouraged when they are misleading due to their ambiguous use in past scientific literature. The general term Extracellular Particles (EPs) is recommended when referring to all particles outside the cells, whether delimited by a lipid bilayer or constituted by an undelimited multimolecular assembly, irrespective of their size or origin.

The application of cell-based therapies or tissues containing regenerative cell populations represents a cutting-edge strategy in regenerative medicine. However, the use of viable whole cells or tissues poses safety concerns and potential risks, including thromboembolism, immune rejection, and tumorigenicity [13–16]. Similarly, the conventional clinical use of hAM tissue patches in ophthalmology, though effective, requires surgical implantation (often with sutures or glues) and can temporarily compromise visual acuity due to graft opacity. In this context, EVs derived from hAM-associated cellular sources represent promising “cell-free” therapeutic agents.

In terms of safety, EVs exhibit markedly lower immunogenicity and tumorigenic potential, and their nanoscale size minimizes the risk of embolic complications. Moreover, regarding clinical translation, EVs offer superior versatility. Unlike the whole tissue graft, they can be formulated as non-invasive topical treatments, such as eye drops — a therapeutic strategy whose feasibility is now widely recognized as a key area of research for ocular surface regeneration [17]. This approach is supported by high-impact preclinical models [18] and topical EV preparations from other MSC sources have already been evaluated in a randomized clinical trial [19], bypassing the need for surgical intervention and preserving corneal transparency.

Crucially, EVs derived from hAM or human amniotic fluid (hAF) also possess intrinsic immunomodulatory properties. For example, they can suppress T-cell proliferation and activation, primarily due to the expression of non-classical HLA molecules such as HLA-G and HLA-E [20, 21]. These features contribute to their low immunogenicity and support a more favorable immunological profile compared to whole-cell therapies. Furthermore, looking toward future applications, EVs offer a platform for bioengineering for targeted delivery, and their molecular cargo can be selectively modulated to enrich therapeutic molecules, thereby enhancing their efficacy [22].

## Potential physiological functions of the EVs isolated from hAM

EVs derived from the hAM and hAF undergo dynamic changes in molecular composition and functional properties throughout gestation, reflecting their critical roles in fetal development and maternal-fetal communication [23–26]. These EVs originate from diverse sources - including fetal urine, fetal and maternal placental tissue, and amniotic epithelial and mesenchymal stromal cells - and exhibit source-specific surface markers such as CD24, CD133, CD105, and HLA-G [23, 24, 27]. Current research reports that from early stages of pregnancy, EVs may participate in blastocyst attachment and implantation, while through gestation they contribute to immune tolerance, angiogenesis, and tissue remodeling, thereby supporting placental development and fetal growth [28–31]. As suggested in the review by Atukorala and colleagues [24] and supported by experimental findings from the same group in a subsequent recent article [27], EVs deriving from hAF originating from the gestational feto-placental unit undergo pregnancy stage-specific modifications in their composition and function. By term, their cargo adapts to maintain fetal homeostasis and promote final organ maturation [27, 32]. It is crucial to note, however, that these conclusions, while intriguing, are drawn from an extremely limited body of evidence. Functionally, EVs, likely originating from placental, amniotic and fetal urine sources, carry immunomodulatory molecules, such as HLA-G, FasL and TRAIL, which promote maternal immune tolerance [20, 23, 29, 33]. They also support placental angiogenesis under physiological conditions. However, in pathological conditions like preeclampsia, EVs deriving from hAF display elevated CD105 expression and exhibit antiangiogenic properties. These alterations are associated with the pre-eclampic state and may contribute to the impaired maternal and fetal vascular development observed in this condition [23], although whether they represent a causal mechanism or primarily reflect the underlying pathology remains to be elucidated. Moreover, their cargo includes growth factors, microRNAs (miRNAs), and extracellular matrix (ECM) components that drive fetal tissue morphogenesis and differentiation, with gestational age-dependent modulation [27, 30, 34, 35]. During labor, EVs deriving from both hAM and hAF respond to inflammatory and mechanical cues, potentially modulating uterine and membrane remodeling processes [23, 29]. In pregnancy complications, altered EV cargo and surface markers contribute to pro-inflammatory and antiangiogenic environments, reinforcing their potential utility as disease biomarkers [23, 29].

A key methodological challenge in the field, however, remains the in situ characterization of EV biogenesis and secretion. Recent ultrastructural investigations have described hAM as anatomically and functionally heterogeneous, composed of four distinct regions — central, intermediate, peripheral, and reflected — each characterized by unique epithelial morphology, microvillar architecture, and cytoplasmic organization [36, 37]. Notably, the secretion of EVs was observed exclusively at the apical surface of amniotic epithelial cells within the central region, suggesting spatial restriction in vesicle production and identifying this area as a potential site of local EV-mediated communication [36]. However, in the absence of specific markers identifying maternal EVs, no definitive conclusions can be drawn regarding its role in feto-maternal EV-mediated signaling. In situ transmission electron microscopy enabled localization and morphometric analysis of these vesicles, which exhibited size and morphology consistent with ectosome-like structures and aimed to have avoided artifacts associated with standard isolation protocols [36]. This spatially resolved evidence of vesicle secretion not only highlights the region-specific functionality of hAM but also underscores the importance of anatomical origin in optimizing the therapeutic application of EVs isolated from hAM. While this spatial insight is relevant, the MISEV2023 [11] guidelines caution against asserting biogenesis based only on morphological evidence, in this particular case static Transmission Electron Microscopy images, which is often insufficient to distinguish secretion pathways. This methodological limitation underscores the need for multimodal approaches to fully understand and eventually optimize the possible therapeutic application of EVs isolated from hAM [36].

## Roles of EVs isolated from hAM in inflammation and fibrosis

The hAM secretome has been extensively investigated for its potential therapeutic effects in inflammation and fibrosis, primarily characterized in the conditioned medium (CM) of either hAM epithelial (hAECs) or mesenchymal stromal cells (hAM-MSCs), analyzed independently. Ragni E. and colleagues conducted a comprehensive characterization of the hAM-MSC secretome, including both the total secretome and EV fraction, identifying a wide array of soluble factors and EV-associated molecules [35]. Their analysis revealed multiple miRNAs and soluble factors involved in cell chemotaxis, inhibition of inflammation, and immune modulation. Functionally, they demonstrated that the complete secretome was capable of downregulating key inflammatory mediators such as cyclooxygenase-2, matrix metalloproteinases, and type I collagen in inflamed chondrocytes and tenocytes. Although these functional findings highlight the therapeutic potential of the hAM secretome, the specific contribution of the EV fraction to these effects remains to be elucidated. Overall, these findings suggest several types of mechanisms through which the hAM secretome may mitigate inflammatory damage and prevent fibrotic progression.

Subsequently, the immunomodulatory properties of both the EV-free and EV-enriched fractions of CM derived from hAM-MSCs were investigated. Notably, the results demonstrated that only the soluble fraction exerted immunomodulatory effects on peripheral blood mononuclear cell (PBMC) activity. This highlights the complexity of the hAM secretome, wherein distinct components may elicit cell type-specific and context-dependent responses [6].

Conversely, other studies have reported significant therapeutic efficacy of the EV fraction alone in models of solid tissue fibrosis. Anti-inflammatory and antifibrotic effects were observed in mouse and rat models of chronic liver fibrosis using EVs derived from both hAM-MSCs and hAECs [38, 39]. Supporting these findings, Mao Y. et al. provided further mechanistic insight, demonstrating that EVs derived from hAM are essential for anti-fibrotic activity [40]. Specifically, they showed that EVs directly suppress the activation and migration of fibroblasts (hepatic stellate cells) and downregulate TGF-β-induced pro-fibrotic markers (α-SMA, COL1A1), an effect not achievable by the EV-depleted secretome. Conversely, Tan J.L. et al. showed that EVs from hAECs promote the polarization of macrophages from pro-inflammatory (M1) to pro-regenerative (M2) phenotype, thereby resolving inflammation and limiting fibrosis [41]. This dichotomy suggests that while soluble factors may drive acute immunomodulation in circulating immune cells like PBMCs, EVs might be more effective in targeting tissue-resident cells, like macrophages and fibroblast, to modulate tissue remodeling signaling, likely through the delivery of miRNAs, lipids, and proteins that regulate fibrogenic and immune-related signaling pathways.

Collectively, these studies underscore the therapeutic potential of the hAM secretome, which appears to act through a sophisticated interplay between soluble factors, EVs, and ECM components, whose relative contribution may vary depending on the cellular source and biological context. This complexity, involving soluble mediators, EVs, and ECM components, offers promising applications in the treatment of a wide range of inflammatory and fibrotic disorders. While most data are derived from non-ocular models, these mechanisms of anti-fibrotic and anti-inflammatory regulation are of paramount interest for ophthalmology, particularly in managing corneal scarring and post-surgical fibrosis. However, further mechanistic studies are needed to elucidate the specific molecular cargo of EVs responsible for these effects and to optimize their application as cell-free therapeutic agents.

## Role of EVs isolated from hAM in ocular diseases

Many common ocular diseases share converging pathogenic mechanisms, including oxidative stress, chronic inflammation, dysregulated angiogenesis, and progressive neurodegeneration, all of which contribute to the structural and functional deterioration of ocular tissues [42]. These pathological processes represent rational therapeutic targets for EVs. A recent retrospective AI-based analysis of publication, funding, and clinical trial data [43] reports that ophthalmology, although of paramount interest as a field for EVs based therapies, is not currently experiencing the same strong acceleration in research seen in other areas. As underscored, while constant growth in research is evident by the publication number, there are only few trials ongoing in the field of ophthalmology. Authors state that filling the translational gap requires a better understanding of EV biology and functions in the eye, both to identify disease-associated cellular alterations and to develop effective EV-based therapeutic strategies. As for the interventional applications of the EVs, hAM represents a valid option as a source of EVs for several reasons: as already mentioned, hAM and its resident cell populations produce EVs whose bioactive cargo exerts antioxidant, anti-inflammatory, anti-angiogenic, and neuroprotective effects [4], resembling the effect of the entire tissue employed in eye surgery; the hAM is a broadly available waste tissue characterized by minimal immunotoxicity, low immunogenicity, and low reactogenicity (a limited propensity to induce adverse inflammatory or immune reactions following administration), while also exhibiting immunosuppressive properties [44, 45]. Moreover, MSCs derived from hAM maintain positive immunomodulatory properties even in pathological conditions, such as preeclampsia [46]. In addition, compared to other perinatal tissue sources such as umbilical cord, hAM benefits from a longer history of clinical use in ophthalmology, resulting in a broader body of evidence supporting its safety and therapeutic application in ocular surgery [47, 48]. Although both tissues share similar challenges related to collection, processing and biobanking, the extensive ophthalmic experience with hAM provides an important translational advantage [49]. Although MSC-derived EVs are generally considered to possess low immunogenicity, the potential immunological interactions of EVs from different cellular sources remain an active area of investigation, highlighting the importance of source selection when developing therapeutic applications [50]. Taken together, these characteristics make hAM an attractive source of EVs for the development of cell-free therapeutic strategies in ophthalmology. Table 1 shows a comparison of the main characteristics of EVs derived from different tissue sources, highlighting clinical potential advantages of extracellular vesicles from human amniotic membrane (hAM-EVs).

**Table 1:** Main characteristics of extracellular vesicles (EVs) derived from different tissue sources [51–56]. MSC - mesenchymal stromal cell; hAM-EV - extracellular vesicle from human amniotic membrane.

Characteristic	hAM-EVs	Bone marrow MSC-EVs	Adipose MSC-EVs	Umbilical cord MSC-EVs
Source availability	Placenta (medical waste)	Bone marrow aspiration	Liposuction	Umbilical cord
Collection invasiveness	None	Invasive	Minimally invasive	None
Ethical concern	Minimal	Moderate	Moderate	Minimal
Clinical experience of source tissue	Extensive in ophthalmology	Extensive in regenerative medicine	Extensive	Increasing
Immunomodulatory potential	Well documented	Well documented	Well documented	Well documented
Need for donor intervention	No	Yes	Yes	Yes
Scalability	High	Moderate	High	Moderate

Given their pleiotropic biological activities, EVs deriving from hAM have been garnering increasing interest as cell-free therapeutic agents in ophthalmology and are now being actively investigated in a few preclinical models.

In age-related macular degeneration (AMD), a leading cause of vision loss in the elderly, characterized by macular degeneration, drusen accumulation, and progressive central vision impairment [50], EVs derived from hAM-MSCs have been shown to protect retinal pigment epithelial cells from oxidative stress both *in vitro* and in murine *in vivo* models. Mechanistically, this protective effect was mediated via activation of the PI3K/Akt/FoxO3 signaling pathway, leading to reduced apoptosis, decreased reactive oxygen species accumulation, and preservation of retinal architecture. Functional benefits included maintenance of retinal thickness and enhanced photoreceptor survival, as demonstrated by histological analysis and TUNEL staining [57].

Regarding glaucoma (a group of optic neuropathies often linked to elevated intraocular pressure and progressive loss of retinal ganglion cells (RGC) [58]), Seong et al. showed that intravitreal injection of CM enriched with EVs from both hAM-MSCs and hAECs resulted in a reduction in intraocular pressure, as measured by tonometry at multiple time points post-injection, in a rat model of glaucoma [59]. Immunohistochemical analyses and *ex vivo* retinal ganglion cell survival assays confirmed a neuroprotective effect, with decreased apoptotic cell counts and preservation of dendritic morphology in treated eyes [59]. However, as this study utilized EV-enriched CM, distinguishing the specific contribution of vesicular *versus* soluble factors remains an area for future investigations.

Similarly, in models of diabetic retinopathy, pathology driven by hyperglycemia-induced inflammation and breakdown of the blood-retinal barrier, subconjunctival administration of hAM-MSC-derived EV-rich CM delayed the progression of retinal vascular pathology, reduced lens opacity, and alleviated ocular inflammation. These findings underscore the broad spectrum of protective actions mediated by EVs deriving from hAM in ocular diseases with underlying inflammatory and metabolic components [60].

At the ocular surface, EVs derived from hAECs promoted proliferation and migration of corneal epithelial cells subjected to hyperosmolar stress *in vitro*, as evidenced by scratch assays and Ki-67 immunostaining. In a murine model of dry eye disease, a condition marked by tear instability and epithelial damage, topical EV administration improved tear film stability (measured by tear breakup time), reduced corneal fluorescein staining, and enhanced epithelial regeneration. Histological analysis confirmed increased epithelial thickness and a marked reduction in inflammatory cell infiltration [61].

Building upon these ocular surface applications, EVs derived from hAEC have demonstrated high efficacy in treating severe corneal alkali burns, offering a reliable cell-free alternative to traditional amniotic membrane transplantation. *In vivo* studies show that hAEC-EVs accelerate re-epithelialization, reduce corneal opacity, and promote an organized collagen fiber arrangement. High-throughput molecular profiling revealed that the hAEC-EV cargo includes specific miRNAs (such as hsa-miR-23-3p, hsa-miR-27a/b-3p, and hsa-miR-181a-5p) and a core set of 167 conserved proteins (including TGFBI, CD44, FN1, and TIMP2) involved in cell adhesion and matrix organization [62]. Mechanistically, hAEC-EVs restore matrix homeostasis by activating focal adhesion kinase (FAK) signaling, upregulating downstream structural proteins like alpha-actinin, tensin-2, and paxillin, and modulating matrix-related genes. This includes the upregulation of ECM-stabilizing molecules like alpha-2-macroglobulin (A2M), laminin subunit alpha 1 (LAMA1), and vitrin (VIT), alongside the downregulation of the inflammatory protease cathepsin B (CTSB). Furthermore, hAEC-EVs prevent corneal scarring and haze by attenuating the TGF-beta/SMAD2/3 pathway, thereby inhibiting key fibrotic markers such as alpha-SMA and connective tissue growth factor (CTGF). *In vitro*, hAEC-EVs promote FAK-dependent proliferation and migration in both epithelial and stromal cells, while bioinformatic insights suggest they effectively guide the local immune microenvironment toward a regenerative state [63].

Collectively, these preclinical studies suggest that EVs deriving from hAM confer multifaceted protective effects in the eye — including neuroprotection, antioxidative activity, anti-inflammatory effects, and trophic support for epithelial and neural cells. While detailed molecular characterization of the specific cargo responsible for these effects was not performed in all cited studies, the consistent observation of anti-inflammatory and trophic outcomes supports the potential of EVs deriving from hAM as a versatile therapeutic tool in ophthalmology. These therapeutic benefits are likely mediated by the EVs’ bioactive cargo, which may include proteins, soluble trophic or neuroprotective factors, anti-inflammatory mediators, and other signaling molecules. Importantly, the therapeutic effects reported across these studies should be interpreted in light of the different EV isolation and enrichment procedures employed. Ultracentrifugation, affinity-based purification, size-exclusion chromatography, precipitation kits, and filtration/concentration of conditioned media can differ substantially in EV recovery, purity, and the extent to which soluble proteins and other non-vesicular components are retained. This is particularly relevant for studies using EV-enriched CM, where the contribution of soluble factors cannot be clearly distinguished from that of EVs. Consequently, differences in isolation and characterization procedures represent a potential source of technical heterogeneity that may contribute to differences in reported biological effects and limit direct cross-study comparison.

The main preclinical studies investigating the therapeutic effects of hAM-derived EVs in ocular diseases are summarized in Table 2.

**Table 2:** Summary of preclinical studies investigating the therapeutic effects of human amniotic membrane (hAM)-derived extracellular vesicles (EVs) in ocular diseases. MSC - mesenchymal stromal cell; hAEC - human amniotic epithelial cell; CM - conditioned medium; RPE - retinal pigment epithelium; RGC - retinal ganglion cell

Disease	EV source and isolation methods	Experimental model	Main findings	Proposed mechanism	Ref.
AMD	hAM-MSC-derived EVs Ultracentrifugation	Human RPE cells; *in vivo* AMD mouse model	Reduced oxidative stress and apoptosis; preserved retinal architecture	PI3K/Akt/FoxO3 pathway activation	[57]
Glaucoma	hAM-MSC and hAEC EV- enriched CM Filtration and concentration of CM	Isolated rat RGCs and human RPE cells; *in vivo* hyperosmotic rat glaucoma model	Reduced intraocular pressure; RGC apoptosis; preserved dendritic morphology	Neuroprotection	[59]
Diabetic retinopathy	hAM-MSC-derived EV-rich CM Filtration and concentration of CM	Streptozotocin-induced diabetic rat model	Delayed retinal vascular damage; reduced lens opacity; alleviated ocular inflammation	Anti-inflammatory and vasculoprotective effects	[60]
Dry eye disease	hAEC-derived EVs Ultracentrifugation and affinity-based EV purification	Isolated human corneal epithelial cells; dry eye mouse model	Enhanced epithelial proliferation and migration; improved tear film stability; reduced corneal damage and inflammatory infiltration	Pro-regenerative and immunomodulatory effects	[61]
Corneal injury	hAEC-derived EVs Ultracentrifugation	*In vitro* corneal epithelial and stromal cells; *in vivo* rabbit corneal alkali burn model	Accelerated re-epithelialization; reduced corneal opacity and fibrosis; improved collagen organization; enhanced wound healing	Activation of FAK signaling; restoration of ECM homeostasis; inhibition of TGF-β/SMAD2/3 signaling; modulation of EV miRNA/protein cargo promoting regeneration	[62, 63]

## Discussion

Despite the increasing interest in EV-based therapies, dedicated analyses of EVs derived specifically from hAM remain limited. Existing reviews have mainly addressed the broader therapeutic applications of hAM or EVs from multiple biological sources [64, 65], whereas the specific contribution, molecular characterization, and translational potential of hAM-derived EVs in ophthalmology remain insufficiently explored. This knowledge gap represents the rationale for the present mini-review.

**Fig. 1:**
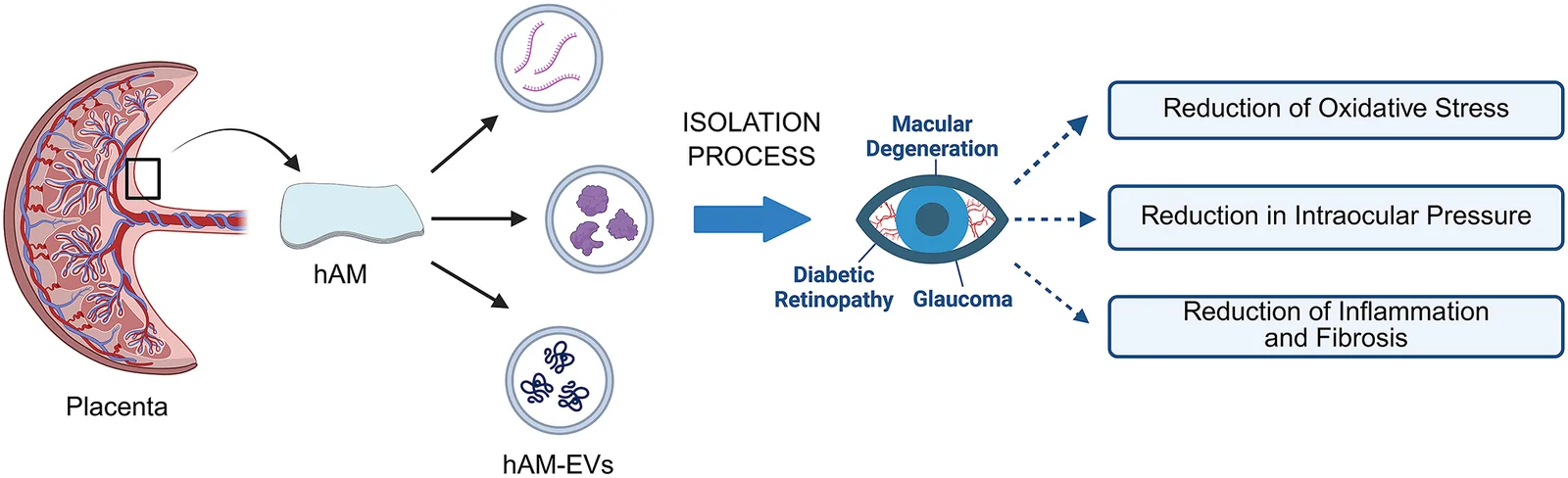
- Potential beneficial effects of extracellular vesicles isolated from human amniotic membrane (hAM-EVs), in human severe eye diseases. hAM represents a valuable source of EVs, endowed with the ability to harbour numerous potentially active biological molecules (proteins, lipids, nucleic acids). Methods for EV isolation and their characterization as therapeutic tools enable their potential application in severe retinal diseases, such as diabetic retinopathy, glaucoma, and macular degeneration. The potential therapeutic actions include the reduction of oxidative stress, the decrease of intraocular pressure, and the modulation of inflammatory processes, leading to reduced fibrosis (created with BioRender.com, https://app.biorender.com/, accessed on 15 of September, 2025)

EVs deriving from hAM represent an emerging cell-free therapeutic platform endowed with immunomodulatory, anti-fibrotic, and pro-regenerative properties (Fig. 1), positioned to overcome some limits of the regenerative medicine. As synthesized in this mini-review, beyond their general properties as a carrier, hAM-EVs seem to recapitulate many of the beneficial effects historically associated with the intact amniotic membrane, while potentially offering greater flexibility and standardizability.

It is tempting to speculate that EVs deriving from hAM could enhance the positive effects observed in hAM engraftment not attributable to the simple mechanical properties of the tissue, or that they could substitute for whole extracts when a pharmacological effect is required. Moreover, compared with most adult tissue sources of EVs, hAM offers greater scalability as starting material for vesicles preparation, owing to its abundance, broad availability as routinely discarded perinatal tissue, and the relative ease of procurement, processing, and storage.

However, transitioning from promising preclinical observations to clinical reality requires a critical reassessment of the current state of the art. While results in ocular models, ranging from corneal regeneration to retinal neuroprotection, are encouraging, the field is currently constrained by the methodological heterogeneity highlighted throughout this manuscript. To address these limitations, critical challenges regarding manufacturing standardization, potency assessment, reproducibility, safety, and regulatory pathways must be resolved.

A primary hurdle lies in standardization and batch-to-batch reproducibility. Since hAM is a biological tissue, donor-to-donor variability, gestational age, and regional tissue heterogeneity can result in preparations with variable purity and biological activity, impairing cross-study reproducibility. For example, it has been observed that amniotic membrane-derived EVs differ in size depending on whether they are isolated from the conditioned medium of whole tissue or from amniotic mesenchymal cells [40]. Furthermore, as emphasized by the MISEV2023 guidelines, much of the existing literature relies on incomplete characterization, making it difficult to attribute specific functions to defined EV subpopulations *versus* co-isolated soluble factors [11]. Importantly, to the best of our knowledge, no Good Manufacturing Practice (GMP)-grade manufacturing protocol has been reported for hAM-derived EVs specifically. Preparatory work in this direction does exist: native characterization and quality-control profiling of hAM-MSC vesicular fractions have been described [8], and molecular fingerprinting of the hAM-MSC secretome has been proposed as a basis for developing potency and release assays for clinical-grade batches [35]. At the tissue level, perinatal sources have been compared under GMP conditions, showing feasible yields but also appreciable differences in microbial burden between amniotic fluid and placental tissues [66]. Validated GMP processes and release matrices are, by contrast, already established for EVs from other MSC sources, including bone marrow and umbilical cord, and have supported regulatory approval of EV-enriched secretomes for a first-in-human trial for the treatment of heart failure [67–69]. The gap for hAM-EVs therefore appears to lie not in technical feasibility, but in the fact that these standards have yet to be applied to this tissue.

Closely related to this, establishing robust potency assessments remains a major translational bottleneck. Because the precise mechanism of action and the synergistic contribution of the vesicular cargo (miRNAs, proteins, lipids) are not fully elucidated, defining validated functional assays to predict *in vivo* efficacy is remarkably complex. This difficulty is compounded by heterogeneity of the preparations themselves, since assays developed for purified EVs may not be transferable to EV-enriched CM, and vice versa. Beyond manufacturing, the rational design of delivery strategies is constrained by a limited understanding of EV biodistribution, cellular uptake mechanisms and long-term fate within tissues [70, 71]. Topically administered EVs, for example as eye drops, may have limited ocular bioavailability because of tear turnover and nasolacrimal drainage, together with the barrier properties of the corneal epithelium [71]. These limitations are particularly important when the posterior segment of the eye needs to be targeted. Invasive approaches, including subconjunctival and intravitreal administration, can improve the access of EVs to ocular tissues, but they also face limitations related to tissue distribution and clearance and may require repeated administration. In addition, intravitreal injection can be associated with procedure-related complications, including increases in intraocular pressure, vitreous opacification, and local inflammatory responses [71, 72].

Several strategies are being investigated to improve EV delivery and retention in ocular tissues. The incorporation of EVs into biomaterial platforms, including hydrogels, may provide a local depot that prolongs EV retention and allows a more controlled delivery [73]. Another approach is the modification of the EV surface to improve its interaction with specific target cells or tissues. This can be achieved, for example, through ligand conjugation or other forms of membrane engineering [70, 73]. The combination of EV engineering with biomaterial-based delivery systems may therefore represent a useful approach to improve ocular EV therapy. However, several issues, including manufacturing reproducibility, formulation, dosing, pharmacokinetics, biodistribution, safety, and long-term therapeutic efficacy, still need to be addressed before these approaches can be translated into clinical applications [70, 71, 73].

From a safety perspective, although hAM tissue has a long-established record of clinical use in ophthalmic surgery, this safety experience cannot be directly extrapolated to purified hAM-EVs, particularly when administered intraocularly. EV preparations may potentially induce local inflammatory responses or exert off-target effects depending on their cellular source, molecular cargo, manufacturing process, and route of administration. Therefore, clinical translation will require rigorous quality control of clinical-grade hAM-EV preparations, including batch-specific endotoxin testing, immunogenicity screening, assessment of potential non-vesicular contaminants, and monitoring of batch-to-batch variability. Finally, long-term safety profiles—including potential risks of altered intraocular pressure or localized immune reactogenicity within the delicate ocular microenvironment—must be thoroughly evaluated. From a regulatory standpoint, international agencies (such as the EMA and FDA) classify unmodified EVs as complex biological medicinal products rather than traditional drugs [74]. Implementing rigorous quality control criteria that comply with these evolving frameworks is a mandatory prerequisite to safely translate these inherently bioactive vesicles into validated clinical treatments.

Looking forward, future research efforts must converge on defining rigorous standards. First and foremost, the adoption of MISEV-compliant protocols is mandatory to clearly define the “active pharmaceutical ingredient” and resolve the persisting ambiguity between epithelial, mesenchymal, and fluid-derived sources [75]. This standardization is a prerequisite for developing validated functional assays, capable of correlating specific molecular cargo with therapeutic outcomes, thereby mitigating the unavoidable impact of donor-to-donor variability [76]. Finally, achieving true translational rigor will require moving beyond small-animal models toward large-animal studies that more accurately replicate the human ocular anatomy and immune response.

## Conclusions

In summary, hAM-derived EVs represent a compelling frontier in regenerative ophthalmology, offering multifunctional therapeutic potential and a viable, cell-free alternative to current treatment modalities. While the path to clinical translation requires navigating the methodological and biological hurdles discussed, the potential reward is significant. As the field moves toward rigorous standardization and functional validation, EVs derived from hAM hold the promise of moving beyond symptomatic management toward promoting intrinsic tissue repair and regeneration. With its unique anatomical accessibility, ophthalmology stands to be one of the primary beneficiaries of this translational success, turning the biological potential of the amniotic secretome into a tangible clinical reality.

## Data Availability

No datasets were generated or analysed during the current study.

## Abbreviations

hAM: Human amniotic membrane
EV: Extracellular vesicles
MISEV: Minimal Information for Studies of Extracellular Vesicles
ISEV: International Society for Extracellular Vesicles
EP: Extracellular Particle
hAF: Human amniotic fluid
CM: Conditioned medium
hAEC: Human amniotic epithelial cell
hAM: MSC-human amniotic mesenchymal stromal cell
MSC: Mesenchymal stromal cell
PBMC: Peripheral blood mononuclear cell
AMD: Age-related macular degeneration
TUNEL: Terminal deoxynucleotidyl transferase dUTP nick end labeling
ECM: Extracellular matrix
FAK: Focal adhesion kinase
A2M: Alpha-2-macroglobulin
LAMA1: Laminin subunit alpha 1
VIT: Vitrin
CTSB: Cathepsin B
CTGF: Connective tissue growth factor
RPE: Retinal pigment epithelium
RGC: Retinal ganglion cell
GMP: Good Manufacturing Practice
EMA: European Medicines Agency
FDA: Food and Drug Administration
hAM-EV: Extracellular vesicle from human amniotic membrane
